# Supplementary material for: “What can her body do?” Reducing weight stigma by appreciating another person’s body functionality
Source: PLoS One. 2021 May 14;16(5):e0251507. doi: 10.1371/journal.pone.0251507 (PMC8121301; doi:10.1371/journal.pone.0251507)
Supplement: S1 File — (DOCX) [file pone.0251507.s001.docx]

**Experimental Manipulations: Writing Exercise Instructions**

This Supporting Information document (S1 File) contains the following: (a) the instructions for the writing exercise given to the experimental group (p. 2); and (b) the instructions for the writing exercise given to the active control group (p. 6). The formatting of these writing exercise instructions is identical to how it was presented to the participants.

**Imagination Assignment: Introduction**

To practice your imagination skills, we would like you to complete the following writing exercise.

In this writing exercise, you will be writing about the woman in the photograph next to you, named ***Anne***.

Specifically, we would like you to imagine, and write about, the many things that her body is able to ***do.***

Below is a list of some possible body functions. Please read through these functions now.

Body functions related to bodily senses and sensations:

Sight

Taste

Touch

Hearing

Smell

Experience pleasure

Feel emotion

Sex drive

Body functions related to physical capacities:

Running

Jumping

Walking

Stretching

Flexibility

Physical coordination

Agility

Balance

Strength

Stamina

Energy level

Reflexes

Sports (e.g., soccer, swimming, zumba)

Yoga

Climbing

Cycling

Body functions related to internal processes:

Healing from a cold

Digesting food

Absorbing vitamins

Creating a baby

Healing from a wound

Growing (hair, nails, new skin cells, etc.)

Regulating temperature, hunger, thirst, etc.

General restoration (e.g., during sleep)

Removing toxins from the body (e.g., through the liver)

Breathing

Body functions related to creative endeavors:

Dancing

Painting

Drawing

Building

Sculpting

Carving

Writing

Singing

Playing an instrument

Reading

Photography

Gardening

Body functions related to self-care:

Sleeping/napping

Eating

Drinking

Cooking

Caring for the body (e.g., by showering or taking a bath)

Body functions related to relationships with others and communication:

Talking

Body language

Facial expressions (e.g., smiling)

Sexual activities

Hugging

Cuddling

Kissing

Crying

Shaking hands

Making eye contact

Being a shoulder to cry on

Giving (or receiving) a massage

Writing a letter

**Imagination Assignment: Description of the Task**

In the Word document on the computer screen, we would like you to **write about the functions of Anne’s body**. We would like you to take your time, really let go and imagine the many different things that her body **can do**. You may focus on as many body functions as you like, but we would like you to try and **consider the different categories** of body functions. You may refer to the list of body functions for inspiration (see booklet on the table). In addition, when you are writing about Anne’s body functions, please **reflect on what these functions might mean to her.** Ask yourself, “Why are these functions important to Anne?”

We will give you **15 minutes** to work on this writing exercise. Do not worry about “finishing” the writing exercise. Just write as much as you can in 15 minutes. Once you have started writing, **do not stop** until the 15 minutes have passed. We will notify you as soon as your 15 minutes are up.

Your writing will be completely confidential and anonymous. Don’t worry about spelling, sentence structure, or grammar. Lastly, there are no right or wrong answers. Your writing will be unique depending on **your imagination.**

Now, go ahead and get started!

**Imagination Assignment: Introduction**

To practice your imagination skills, we would like you to complete the following writing exercise.

In this writing exercise, you will be writing about the woman in the photograph next to you, named ***Anne***.

Specifically, we would like you to imagine, and write about, the ***house*** in which Anne lives, and the ***details*** of her house.

Below is a list of some possible features you could describe. Please read through these features now.

Types of houses:
Apartment, studio, or loft
Condominium
Townhome
Cottage

Bungalow

Villa

Exterior of the house:
Doors

Windows

Shutters

Front porch

Materials (e.g., wood, stone)

Flower boxes

Mailbox

Outdoor space:
Lighting
Garage
Driveway
Pathway
Greenery (e.g., plants, trees, flowers)

Decorative features (e.g., fountain, statue)

Fences/gates

Structure of the house:
Number of floors
Number of rooms
Types of rooms (e.g., bathroom, bedroom)
Terrace or balcony
Hallways

Stairs

Interior design of the house:
Minimalist

Scandinavian

Country/rustic

Modern

Eclectic

Bohemian

Industrial

Nautical

Furnishings and decor:
Tables

Chairs

Lighting

Cupboards/shelves

Carpets/rugs

Imagery (e.g., paintings, pictures, posters)

Soft furnishings (e.g., blankets, pillows, cushions)

Greenery (e.g., plants, flowers)

**Imagination Assignment: Description of the Task**

In the Word document on the computer screen, we would like you to **write about the house in which Anne lives.** We would like you to take your time, really let go and imagine the many different details of **her house**. You may focus on as many features as you like, but we would like you to try and **consider the different categories** of home features. You may refer to the list of features for inspiration (see booklet on the table).

We will give you **15 minutes** to work on this writing exercise. Do not worry about “finishing” the writing exercise. Just write as much as you can in 15 minutes. Once you have started writing, **do not stop** until the 15 minutes have passed. We will notify you as soon as your 15 minutes are up.

Your writing will be completely confidential and anonymous. Don’t worry about spelling, sentence structure, or grammar. Lastly, there are no right or wrong answers. Your writing will be unique depending on **your imagination.**

Now, go ahead and get started!
